# Association between exercise self-efficacy and physical activity in elderly individuals: a systematic review and meta-analysis

**DOI:** 10.3389/fpsyg.2025.1525277

**Published:** 2025-06-10

**Authors:** Lei Xie, Wenxue Ma, Kangli Du, Ying Huang, Aihua Li, Hongwei Wang, Hongcheng Cui, Wentao Qiu, Rong Gao, Guofeng Qu, Xishuai Wang, Cong Liu

**Affiliations:** ^1^College of Physical Education and Sports, Beijing Normal University, Beijing, China; ^2^College of Education for the Future, Beijing Normal University, Zhuhai, China; ^3^School of Physical Education and Sports Science, Qufu Normal University, Qufu, China; ^4^College of Physical Education, Northwest Normal University, Lanzhou, China; ^5^Leisure and Digital Sports College, Guangzhou Sports University, Guangzhou, China

**Keywords:** exercise self-efficacy, physical activity, elderly, correlation, meta-analysis

## Abstract

**Objective:**

In an aging population, the problem of insufficient physical activity among the elderly is increasingly recognized. Exercise self-efficacy, a critical determinant of physical activity in this demographic, has garnered increasing attention recently. This review focuses on healthy older adults, systematically reviewing the research progress on the relationship between exercise self-efficacy and physical activity in later life. It analyzes the correlation between the two factors and their influencing factors, and explores the mechanism of exercise self-efficacy in promoting physical activity among the elderly.

**Methods:**

In accordance with the standards set by the Preferred Reporting Items for Systematic Reviews and Meta-Analyses (PRISMA) statement, an extensive literature search was conducted across five electronic databases: Web of Science, PubMed, ProQuest, Scopus, and EBSCOhost. The search period spanned from January 1, 2000, to October 20, 2024. A rigorous quality assessment was performed on the selected studies, with methodological and outcome data extracted via a standardized data extraction form. The meta-analysis of the included studies was conducted via Stata 18 software, along with tests for between-study heterogeneity and an evaluation of publication bias.

**Results:**

The literature screening process yielded 19 studies that provided data on the correlation between physical activity and exercise self-efficacy. These studies employed Pearson correlation analysis (15 studies), multiple regression analysis (6 studies), and structural equation modeling (SEM) (4 studies). A random-effects model was used to pool the effect sizes, revealing an average correlation coefficient of *r* = 0.412 (*p* < 0.001). The average standardized coefficient for the effect of exercise self-efficacy on physical activity was *β* = 0.386 (*p* < 0.001), and the average path coefficient for the effect of physical activity on exercise self-efficacy was *γ* = 0.481 (*p* < 0.001).

**Conclusion:**

A significant positive correlation was found between exercise self-efficacy and physical activity among elderly individuals, with a moderate degree of influence of exercise self-efficacy on physical activity participation. Conversely, physical activity also positively impacts exercise self-efficacy in elderly individuals. These findings provide a theoretical basis for encouraging elderly individuals to engage in physical activities and enhance their quality of life. Future research should further investigate the roles of various influencing factors and develop targeted intervention strategies to promote more active participation in physical activities among elderly individuals.

## 1 Introduction

The aging of the global population has brought the issue of insufficient physical activity among the elderly to the forefront. Studies indicate that appropriate physical activity can significantly enhance the health and well-being of older adults, prevent chronic diseases, and improve quality of life (Sun et al., 2013). However, many1 elderly individuals exhibit low levels of physical activity due to various factors. According to the 2020 World Health Organization (WHO) guidelines, only a fraction of older adults engage in regular physical exercise, particularly in multicomponent activities focused on functional balance and strength training (WHO, 2020). Consequently, strategies to increase physical activity levels in elderly individuals have become a research priority. In this context, exercise self-efficacy, as a pivotal psychological factor in promoting physical activity among elderly individuals, has gained attention. Exercise self-efficacy reflects an individual’s confidence in participating in and adhering to physical exercise, a concept rooted in Bandura’s self-efficacy theory, which emphasizes that individual behavior is influenced by beliefs about one’s capabilities and anticipated outcomes (Bandura and Wessels, 1997). This systematic review focuses on individuals aged 60 and above to align with the World Health Organization’s definition of older adults and to address the unique health challenges faced by this population. This systematic review aims to uncover the psychological motivation mechanisms underlying elderly individuals’ participation in physical activity by examining the association between exercise self-efficacy and physical activity, providing a scientific basis for developing effective intervention strategies to enhance physical activity engagement and overall health in elderly individuals.

While the association between self-efficacy and physical activity has been extensively studied in adolescents and adults, few review studies have focused on the elderly population. For example, Craggs et al. (2011) noted in their review on determinants of physical activity change in children and adolescents that self-efficacy was related to physical activity changes in older children. Similarly, in their review on physical fitness, exercise self-efficacy, and quality of life in adulthood, Medrano-Ureña et al. (2020) found that the majority of studies reported a significant correlation between exercise self-efficacy and physical activity. This research bias may have led to an underestimation of the importance of self-efficacy in strategies to promote physical activity among elderly individuals. Given the distinct physiological and psychological developmental stages of adolescents and adults, their mechanisms of self-efficacy formation and function may significantly differ from those of elderly individuals. Physical activity participation in the elderly is often influenced by physiological constraints, chronic diseases, and changes in social roles, making it crucial to conduct research specific to the elderly to understand their unique needs and challenges in physical activity.

Previous research has fallen short in addressing the relationship between self-efficacy and the general healthy population, with a majority of studies focusing on pathological groups. For example, Hamidi et al. (2022) reported a significant association between self-efficacy and physical activity in diabetic patients. Similarly, Selzler et al. (2020) noted a positive correlation between self-efficacy and functional exercise capacity as well as physical activity in individuals with COPD, although the strength of this association is influenced by the choice of measurement tools. Current review studies predominantly focus on general self-efficacy, with limited attention given to exercise self-efficacy, potentially overlooking its unique role in facilitating physical activity. General self-efficacy pertains to an individual’s overall confidence in facing challenges, whereas exercise self-efficacy is more specific, relating to the confidence level in engaging in sports activities (Bandura, 1997). Given that exercise self-efficacy directly impacts the selection, persistence, and effort invested in sports activities (McAuley et al., 2006), research targeting this domain is essential for developing precise intervention strategies for promoting physical activity among elderly individuals.

The innovation of this study lies in the fact that it is the first systematic review and meta-analysis specifically focusing on healthy elderly individuals to explore the bidirectional relationship between exercise self-efficacy and physical activity. Previous reviews, such as those by Selzler et al. (2020) and Hamidi et al. (2022), primarily targeted clinical populations (e.g., individuals with chronic obstructive pulmonary disease or diabetes mellitus), whereas this study focuses on healthy older adults. Furthermore, it is the first to integrate effect sizes from various statistical methods, including correlation coefficients, regression coefficients, and path coefficients from structural equation modeling. This methodological innovation allows for the quantification of between-study variations in results, providing more robust scientific evidence for developing interventions to promote physical activity among elderly individuals.

This systematic review aims to examine the association between exercise self-efficacy and physical activity in elderly individuals, uncover the psychological mechanisms underlying their participation in physical activity, and provide a scientific basis for developing targeted interventions to enhance physical activity engagement, improve health outcomes, and address the global challenge of aging populations.

## 2 Method

### 2.1 Literature search strategy

This study adheres to the PRISMA 2020 Statement (Page et al., 2021) and systematically documents the entire literature search process. The search covered five databases: Web of Science, PubMed, ProQuest, Scopus, and EBSCOhost, with the search period ranging from January 1, 2000, to October 20, 2024. The search criteria were set to include documents in which the title or abstract contained ‘physical activity’ OR ‘exercise’ OR ‘circuit training’ OR ‘resistance training’ OR ‘aerobic training’ OR ‘leisure-time activity’ OR ‘sport participation’ OR ‘exercise therapy’ OR ‘sports’ OR ‘physical fitness’ OR ‘swimming’ OR ‘walking’ OR ‘water exercise’ OR ‘power training’ OR ‘muscle stretching exercise’ AND ‘exercise self-efficacy’ OR ‘self-efficacy’ OR ‘self-efficacy beliefs’ OR ‘self-efficacy levels’ AND ‘elderly’ OR ‘senior citizens’ OR ‘aged 80 and above’ OR ‘octogenarian’ OR ‘nonagenarian’ OR ‘centenarian’.

This study only included English—language peer-reviewed literature published from January 1, 2000 to October 20, 2024. This period saw key development in studying the relationship between elderly exercise self—efficacy and physical activity. Significant progress was made in theory construction, measurement tool development, and research—method innovation. This ensured the included literature’s theoretical, methodological, and tool—related advancement and scientific rigor, offering high—quality data for the systematic review and meta—analysis. Limiting the literature—source platform and time—span boosted data reliability and authority, enhancing the research results’ generalizability and academic worth.

The research team’s English proficiency enabled accurate data extraction and ensured the reliability of the study’s findings. English, as the main language of international academic exchange, encompasses a vast number of high-quality research results. However, this choice of excluding non-English studies might limit the general applicability of the research outcomes. Future research could enhance the comprehensiveness of its results by incorporating studies in multiple languages.

### 2.2 Inclusion and exclusion criteria

The inclusion criteria were as follows: (1) participants aged 60 years and older, with an average age exceeding 65 years; (2) studies that included an assessment of exercise-related self-efficacy; (3) research involving an evaluation of physical activity; (4) quantitative analysis of the association between exercise-related self-efficacy and physical activity; (5) studies that were cross-sectional, longitudinal, or long-term; and (6) publications in English.

The exclusion criteria were as follows: (1) This study only included healthy elderly individuals, excluding those with specific diseases such as cardiovascular disease or diabetes. The subjects were defined as individuals without major chronic diseases or health issues; (2) studies not published in peer-reviewed journals; (3) studies with a sample size less than 50; (4) studies that did not provide data on the association between exercise-related self-efficacy and physical activity; and (5) review or regression analysis articles. To ensure the rigor and relevance of this systematic review and meta-analysis, specific inclusion and exclusion criteria were established. We focused on individuals aged 60 years and older with an average age exceeding 65 years, as this population represents the senior demographic with distinct physiological and psychological characteristics regarding physical activity participation. Studies involving specific disease populations such as those with cardiovascular conditions or diabetes were excluded because these groups may have unique restrictions and motivations for physical activity that differ from the general healthy elderly population.

Cross-sectional, longitudinal, and long-term studies were included to capture the complex temporal relationships between exercise self-efficacy and physical activity. Cross-sectional studies provide a snapshot of the association at a single point in time, while longitudinal and long-term studies allow for the examination of causal relationships and changes over time. Only peer-reviewed studies were considered to guarantee the scientific quality and credibility of the included research, as the peer-review process involves rigorous evaluation of study design, data collection, and analysis methods by experts in the field.

Studies with samples of less than 50 were excluded for statistical reasons. Small samples are more likely to have sampling errors, which can make effect size estimates inaccurate and lower statistical power. While removing such studies helps ensure the meta—analysis results on the exercise self—efficacy and physical activity relationship are reliable and valid, it might also exclude studies with extreme results, affecting funnel plot symmetry.It is important to acknowledge that the exclusion of studies with a sample size less than 50 may have influenced the results of this meta-analysis. While this criterion was applied to reduce statistical heterogeneity and enhance the reliability of the findings, it is possible that some studies with smaller sample sizes but potentially significant results were excluded. This could have affected the symmetry of the funnel plot and potentially introduced bias. Future research should consider incorporating studies with smaller sample sizes to provide a more comprehensive understanding of the relationship between exercise self-efficacy and physical activity in elderly individuals.

After de-duplication of the retrieved literature, two researchers independently screened the studies on the basis of the inclusion and exclusion criteria. Initially, a preliminary screening was conducted through titles and abstracts to identify potentially relevant studies for full-text review. While confirming studies for full-text review, a meticulous examination of the reference lists of the obtained full-text articles and other systematic reviews was subsequently conducted to ensure that no eligible studies were missed. Finally, the included studies were identified by consensus between the two researchers. In the case of discrepancies in the screening results, a third researcher made the final decision.

### 2.3 Data extraction

Data were extracted independently by two authors on the basis of the inclusion criteria, with any discrepancies resolved through consultation. The extracted information included the following: (1) author names and publication year; (2) study location; (3) type of study design; (4) methods for assessing exercise self-efficacy; (5) methods for assessing physical activity; (6) statistical analysis techniques; (7) measures of association (e.g., correlation coefficients, standardized coefficients); (8) key findings of the studies.

### 2.4 Quality assessment of the literature

In this study, one reviewer assessed the quality of the included studies, and another reviewer verified the assessment. The Risk of Bias in Non-randomized Studies—of Exposure [ROBINS - E (Higgins et al., 2024)] tool was used for the risk - of - bias assessment. ROBINS-E assesses the risk of bias in non-randomized exposure studies across multiple dimensions: confounding control (D1), exposure measurement accuracy (D2), participant selection (D3), post-exposure intervention effects (D4), missing data handling (D5), outcome measurement reliability (D6), and selective outcome reporting (D7). Each dimension’s risk of bias was rated as low (+), of some concern (/), or high (−). This comprehensive evaluation helps researchers identify and measure potential study biases, enhancing the reliability and validity of the findings.

### 2.5 Statistical analysis

Each study included in this review provided data on the association between exercise self-efficacy and physical activity in elderly individuals, along with the sample size. For longitudinal studies, we selected baseline association data for analysis; for studies that used different assessment tools, we conducted a meta-analysis to combine the association data. The meta-analysis was performed using Stata 18 software, and the converted effect sizes were analyzed. We used a fixed effects model (I^2^ < 50% and *p* > 0.05) or a random effects model (I^2^ ≥ 50% or *p* < 0.05) based on the results of the heterogeneity test. The level of heterogeneity was assessed using the I^2^ index and categorized as low (I^2^ ≤ 25%), moderate (25% < I^2^ ≤ 50%), or high (I^2^ > 50%) (Liu et al., 2023; Higgins et al., 2003). To detect publication bias, we performed Egger’s test and presented the analysis results in funnel plots.

## 3 Results

### 3.1 Study selection process

**Figure 1:**
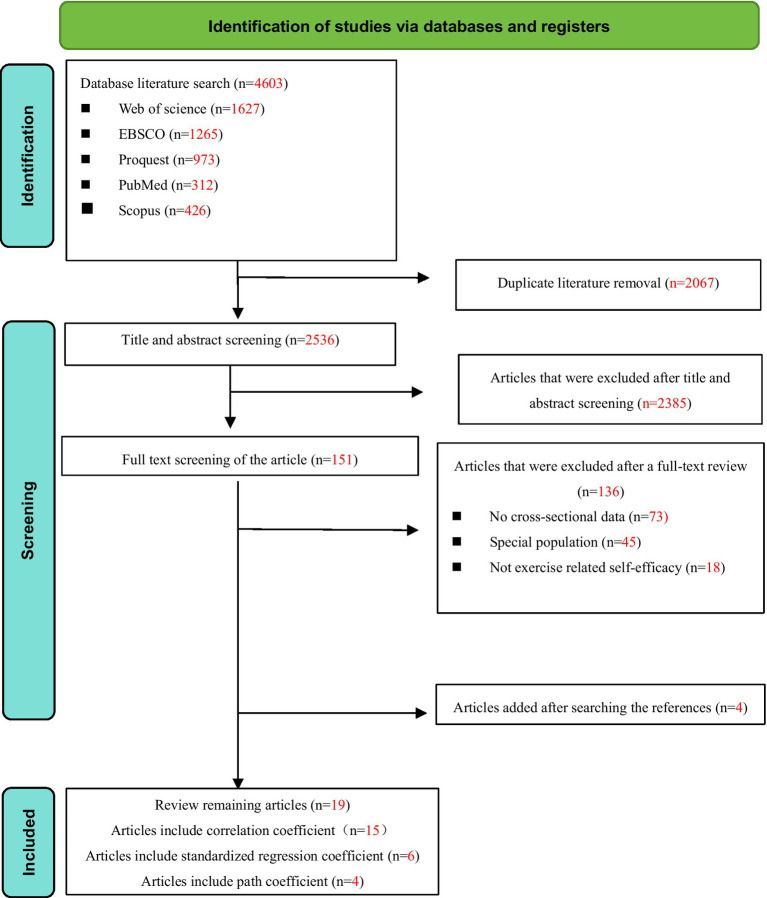
Flow chart of literature screening.

After the initial and full—text screening, 19 studies were included for analysis (see Figure 1). All of them satisfied the inclusion criteria and offered relevant data on the relationship between exercise self—efficacy and physical activity. The database search initially yielded 4,603 potentially relevant articles. After removing duplicates, 2,536 unique articles were screened by title and abstract, with 2,385 being excluded on the basis of the inclusion criteria. A full-text review was conducted for the remaining 151 articles, resulting in the exclusion of 136 articles for various reasons. The primary reasons for exclusion were as follows: 73 articles lacked original data, 45 studies focused on specific populations (e.g., patients with diseases, veterans), and 18 studies did not involve exercise-related self-efficacy. A total of 19 articles met all the inclusion criteria from January 2000 to October 2024.

### 3.2 Basic characteristics of the included studies

Table 1 provides a comprehensive summary of the fundamental details of the 19 studies included in this review. Of the 19 studies incorporated in this review, 68.42% (13 studies) utilized a cross—sectional design, while 31.58% (6 studies) employed a longitudinal design. Regarding the geographical distribution, 57.89% (11 studies) were conducted in the United States, with the remaining 42.11% (8 studies) carried out in the United Kingdom, Spain, Germany, Korea, Indonesia, and Chinese Taipei. The sample sizes of these studies varied, ranging from 71 to 884 participants. Among these studies, 15 conducted correlation analysis and reported correlation coefficients (r). Six studies further employed multiple regression analysis, presenting standardized coefficients (*β*) and standard errors (SE). Furthermore, four studies employed structural equation modeling and reported path coefficients (*γ*) with their 95% confidence intervals. The PRISMA flow diagram (Figure 1) illustrates the process of study selection and the reasons for excluding studies.

**Table 1:** Summary of the included literature.

Number	Author, year	Study design	Country	Sample size	Age (years) mean (SD) [range]	Percentage girls (%)	Instruments used(PA)	Instruments used(ES)	Analysis	Association indicators	Conclusion
1	Langan and Marotta (2000)	CS	US	228	>60	66.2%	PAI-CAQ	PSE	multiple regression	*β* = 0.19 (*p* < 0.05) R^2^ = 0.036	The results of the analysis identified physical activity as the only significant predictor of physical self-efficacy, accounting for 3.6% of the variance in the PSE scores.
2	Laffrey (2000)	CS	US	71	60–87(71.37)	100%	PAQ	S-E	correlations multiple regression	r = 0.5(p < 0.01) β = 0.36 (*p* < 0.01)	Exercise self-efficacy is significantly and positively related to physical activity levels among older Mexican American women, suggesting that enhancing self-efficacy may contribute to promoting greater physical activity engagement in this population.
3	Brassington et al. (2002)	LS	US	103	>65(70.18)	65%	Exercise intervention	SEE	correlations multiple regression	r = 0.46(p < 0.01) *β* = 0.37(*p* < 0.001)	Improving exercise self-efficacy and helping older adults achieve health-related goals are key to promoting exercise adherence.
4	McAuley et al. (2003)	LS	US	174	60–75(66)	74.1%	PASE	BSE ESE	SEM	γ = 0.42	Self-efficacy is a key factor in predicting older adults’ long-term adherence to physical activity and can be enhanced through social support, exercise-related affect, and exercise frequency.
5	McAuley et al. (2005)	LS	US	152/126	60–75(66.7)	72%	PASE	ESE	bivariate correlations	12-month: r = 0.28 60-month: r = 0.09	Physical activity and self-efficacy were related at 1 year, but the relationship between changes in these variables over time was non-*significant. However, the lack of association may be attributed to the manner in which self-efficacy and physical activity were assessed in the present study.
6	McAuley et al. (2007)	LS	US	174	>60 (66.7)	71.8%	PASE	ESE	bivariate correlations	r = 0.32	Being more efficacious and reporting more positive affect at Year 2 were significantly associated with higher levels of activity at both Years 2 and 5.
7	Morris et al. (2008a)	LS	US	137	(69.6)	100%	PASE	BSE	correlation matrix SEM	Baseline: r = 0.261 6-month: r = 0.360 γ = 0.26 (B)	As hypothesized, self-efficacy was directly associated with physical activity.
8	Perkins et al. (2008)	CS	Spain and US	108(53/55)	63–92 (76.7)	58.3%	Exercise frequency inquiry	ESE	multiple regression	Spain: β = 0.391 US: β = 0.486 (p < 0.05)	The finding that self-efficacy is significantly associated with participation in physical activity replicates previous studies that found greater self-efficacy of physical activity led to a higher likelihood of participating in physical activity.
9	Morris et al. (2008b)	CS	US	136	63–75 (69.7)	100%	Actigraph accelerometer	ESE	bivariate correlations multiple regression	r = 0.42 β = 0.29	Among older women, self-efficacy, functional limitations and street connectivity demonstrated independent contributions to physical activity behavior.
10	Harris et al. (2009)	CS	UK	238	≥65	47.9%	Actigraph accelerometer	ESE	linear regression	Moderate (p < 0.05) 1,108 (267 to 1949) High (*p* < 0.05) 1885 (139 to 3,631)	The independent effects of exercise self-efficacy and exercise control on PA levels highlight their roles as potential mediators for intervention studies.
11	Grant-Savela (2010)	CS	US	197	60–96(71.5)	55.3%	PASE	SEE	correlations	r = 0.18 (p < 0.05)	Enhancing older adults’ self-efficacy may boost their frequency and duration of physical activity participation, thereby elevating their overall physical activity levels.
12	Paxton et al. (2010)	CS	US	196	66–82 (74)	75%	GLTEQ	BSE WSE	correlations	r = 0.61	Older adults who participate in physical activity may enhance their self-efficacy beliefs for physical activity and improve their mental health.
13	Warner et al. (2011)	LS	German	309	65–85 (73.3)	42%	Exercise frequency inquiry	ESE	correlations multiple regression	r = 0.40 β = 0.65 R^2^ = 0.28	Support received from friends and exercise self-efficacy were specified as predictors of exercise frequency while baseline exercise, sex, age, and physical functioning were controlled for. In addition to main effects of self-efficacy and social support, an interaction between social support and self-efficacy emerged.
14	Mullen et al. (2012)	CS	US	884	>65 (74.8)	77%	Walking behavior inquiry	WSE	correlations SEM	r = 0.26 γ = 0.36	Walking more frequently and for longer duration was positively associated with beliefs in capabilities of walking incrementally further distances.
15	Mudrak et al. (2016)	CS	Czech	546	≥60 (68)	79.2%	GLTEQ PASE	BSE	correlations SEM	LTEQ/LPASE r = 0.284 PASE/LPASE r = 0.234 γ = 0.808	Physical activity predicted self-efficacy, which in turn predicted global QOL through mental and physical health status.
16	Miller et al. (2019)	CS	Australia	586	65–96 (72.5)	70.6%	CHAMPS	ESE	bivariate correlations	r = 0.44	Exercise behavior (measured as calories per week) is significantly correlated with exercise self-efficacy.
17	Juwita and Damayanti (2022)	CS	Indonesia	106	≥60 (74.4)	60%	GPAQ	ESE	bivariate correlations	r = 0.6	There is a significant correlation between self-efficacy and physical activity in the elderly with a value of r = 0.6, which indicates a positive direction and a strong correlation level.
18	Lee and Fan (2023)	CS	Taiwan	183	≥65 (74.4)	70.73%	PASE	A modified version of a previous scale	bivariate correlations	r = 0.29	Self-rated health and self-efficacy were the significant variables in physical activity for middle-aged and elderly individuals. In addition, neighborhood environment and the interaction between self-efficacy and the neighborhood environment were significant variables in middle-aged adults.
19	Dawe et al. (2024)	CS	US/ITA	726	≥65 (72.57)	51.1%	GLTEQ	ESE	Spearman correlation	r = 0.531	Exercise Self-efficacy has shown a large positive correlation with the leisure score index。

C, Cross-sectional study; LS, Longitudinal study; SEM, Structural Equation Model testing; PAI-CAQ, Physical Activity Index from the College Alumnus Questionnaire; PAQ, Physical Activity Questionnaire; S-E, Self-Efficacy (S-E) questionnaire; LTPA, Leisure Time Physical Activity; PASE, Physical Activity Scale for the Elderly; ESE, Exercise Self-Efficacy scale; WSE, Self-Efficacy for Walking; PSE, Physical Self-Efficacy scale; BSE, Barriers Self-Efficacy scale; SEE, Self-Efficacy for Exercise Scale; GLTEQ, Godin Leisure Time Exercise Questionnaire; QOL, quality of life; CHAMPS, Community Health Activities Model Program for Seniors questionnaire; GPAQ, Global Physical Activity Questionnaire.

### 3.3 Quality assessment of the included studies

The risk of bias assessment results obtained using the ROBINS-E (Higgins et al., 2024) tool. The studies selected for the review showed variability in the different quality assessment domains.

Results – Six studies were found to have a low risk of bias across all analyzed domains. Most studies were judged to have some concerns, due to reasons such as incomplete adjustment for all confounders, issues with exposure measurement, participant selection, post-exposure interventions, missing data, outcome measurement, and selective reporting of results. The overall certainty of the evidence for any specific outcome was not determined, as no indication was provided in the supplied data. For other unmentioned outcomes, we also did not determine the certainty of the evidence due to insufficient information to assess it based on the given data (see Table 2).

**Table 2:** The risk of bias in non-randomized studies—of exposures (ROBINS-E) assessment tool (for follow-up studies).

Study	D1	D2	D3	D4	D5	D6	D7	Overall risk of bias
Langan and Marotta (2000)	/	+	+	+	+	/	+	/
Laffrey (2000)	+	+	/	+	+	+	+	+
Brassington et al. (2002)	/	/	+	/	+	/	+	/
McAuley et al. (2003)	/	/	/	+	/	+	/	/
McAuley et al. (2005)	+	+	/	+	/	+	+	/
McAuley et al. (2007)	+	+	/	+	+	/	+	/
Morris et al. (2008a)	+	+	/	+	/	+	+	/
Perkins et al. (2008)	+	+	/	+	+	/	+	/
Morris et al. (2008a, 2008b)	+	+	/	+	+	+	+	+
Harris et al. (2009)	+	+	+	+	+	+	+	+
Grant-Savela (2010)	+	/	/	+	+	/	+	/
Paxton et al. (2010)	+	+	/	+	+	/	+	/
Warner et al. (2011)	+	+	+	+	+	/	+	+
Mullen et al. (2012)	+	/	+	+	+	/	+	/
Mudrak et al. (2016)	+	/	+	+	+	+	+	+
Miller et al. (2019)	+	+	+	+	+	/	+	+
Juwita and Damayanti (2022)	+	+	/	+	+	/	+	/
Lee and Fan (2023)	+	+	/	+	+	/	+	/
Dawe et al. (2024)	+	+	+	+	+	/	+	+

D1: Risk of bias due to confounding. D2: Risk of bias arising from measurement of the exposure. D3: Risk of bias in selection of participants into the study (or into the analysis). D4: Risk of bias due to post-exposure interventions. D5: Risk of bias due to missing data. D6: Risk of bias arising from measurement of the outcome. D7: Risk of bias in selection of the reported result. Judgement. +: Low risk of bias. /: some concerns. -: High risk of bias.

### 3.4 Assessment tools for physical activity in elderly populations

This study incorporated six different tools to evaluate the frequency and energy expenditure of physical activities among elderly individuals. The Physical Activity Scale for the Elderly (PASE) (Washburn et al., 1993) was utilized in nine studies, providing a comprehensive assessment of daily activities, leisure, and exercise over the past week. Additionally, the Godin Leisure Time Exercise Questionnaire (GLTEQ) (Godin and Shephard, 1985) was applied in four studies, two of which also employed the PASE. The GLTEQ assesses leisure-time physical activity by documenting the frequency and duration of light to vigorous exercise over a year. One study used the Community Health Activities Model Program for Seniors questionnaire (CHAMPS) (Stewart et al., 2001), which spans a range of physical activities from walking to vigorous exercise. The Global Physical Activity Questionnaire (GPAQ) (Armstrong and Bull, 2006), developed by the World Health Organization (WHO), was used in one study to evaluate physical activity across work, travel, and leisure domains. Similarly, the Physical Activity Index from the College Alumnus Questionnaire (PAI-CAQ) (Paffenbarger et al., 1978) was used in one study to assess physical activity levels. Furthermore, two studies employed Actigraph accelerometers to collect objective data on physical activity (Hendelman et al., 2000). An additional four studies (Perkins et al., 2008; Warner et al., 2011; Mullen et al., 2012; Ainsworth et al., 2000) assessed physical activity through inquiries or reports on exercise frequency. These tools offer a multifaceted approach to evaluating physical activity in elderly populations, including the quantification of activity frequency and energy expenditure (see Table 3).

**Table 3:** List of physical activity assessment tools for elderly individuals.

Evaluation tool	Full name	Author, year
PASE	Physical Activity Scale for the Elderly	Washburn et al. (1993)
GLTEQ	Godin Leisure Time Exercise Questionnaire	Godin and Shephard (1985)
PAI-CAQ	Physical Activity Index from the College Alumnus Questionnaire	Paffenbarger et al. (1978)
GPAQ	Global Physical Activity Questionnaire	Armstrong and Bull (2006)
CHAMPS	Community Health Activities Model Program for Seniors questionnaire	Stewart et al. (2001)
Actigraph accelerometer	Actigraph-provided ActiLife Monitoring System	Hendelman et al. (2000)

### 3.5 Assessment of exercise self-efficacy in elderly individuals

The studies included in this review primarily employed four tools to assess the self-efficacy of elderly individuals in engaging in physical activities. The Exercise Self-efficacy scale (ESE) (McAuley, 1993) was used in 10 studies and is widely applied to evaluate individuals’ self-efficacy in maintaining exercise participation. The ESE consists of nine items that measure the confidence level of maintaining a 20-min exercise routine three times a week, despite challenges such as adverse weather, boredom, and physical discomfort. Additionally, two studies utilized the Self-Efficacy for Walking scale (WSE) (McAuley et al., 2000), which focuses on assessing participants’ belief in their walking ability, particularly their ability to successfully complete walks of increasing duration from 5 to 40 min at a moderate pace. The Barriers Self-Efficacy scale (BSE) (McAuley, 1992) was employed in three studies to evaluate individuals’ perceived ability to overcome common barriers to exercise, with the aim of understanding participants’ confidence in maintaining a 40-min exercise routine three times a week over the next 2 months. Finally, the Physical Self-Efficacy scale (PSE) (Bosscher et al., 1993), which includes 10 statements about physical capabilities, was used in two studies, with respondents indicating their agreement or disagreement on a 5-point Likert scale.

### 3.6 Meta-analysis

#### 3.6.1 Meta-analysis of the correlation between physical activity and exercise self-efficacy in elderly individuals

Among the 19 studies included, 15 provided data on the correlation between physical activity and exercise self-efficacy in elderly individuals. To standardize the analysis, Spearman’s rho (*ρ*) coefficients reported in the literature were converted to Pearson’s r coefficients via the following formula: r ≈ 6 sin(*π*ρ/6)/π. These coefficients were then transformed into Fisher’s Z scores for meta-analysis, along with the calculation of standard errors (SE) and 95% confidence intervals (CI) (Borenstein et al., 2011; Szczuka et al., 2021).

**Figure 2:**
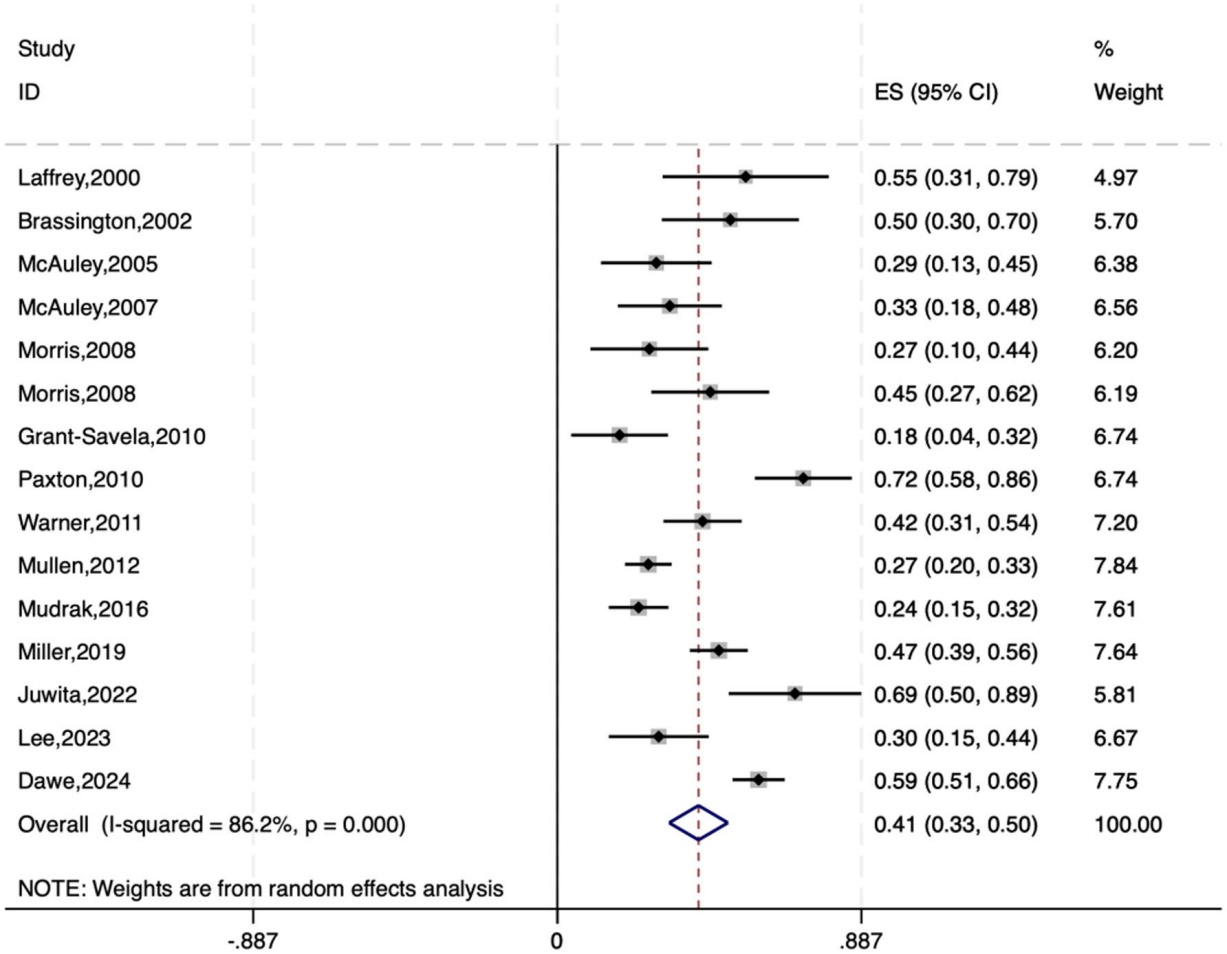
Forest plot of the association between exercise self-efficacy and physical activity among elderly individuals.

A random-effects model was used to conduct the meta-analysis on the correlation coefficients (r values) and their 95% CIs. The results revealed a pooled effect size of 0.412, with a 95% CI ranging from 0.326 to 0.497, indicating a significant overall correlation. The heterogeneity test yielded a chi-square value of 101.45 with 14 degrees of freedom and a *p* value less than 0.0001, suggesting significant heterogeneity among studies. The I-squared value of 86.2% indicates that 86.2% of the variability in effect sizes can be attributed to heterogeneity between studies. Moreover, the z test for an effect size of zero yielded a value of 9.40 with a p value less than 0.0001, further confirming the statistical significance of the findings (see Figure 2).

Converting the pooled Fisher’s Z scores and 95% CIs back to Pearson’s r, the average correlation coefficient was found to be 0.390, with a 95% CI ranging from approximately 0.315 to 0.460. Therefore, the average correlation between physical activity and exercise self-efficacy in elderly individuals is 0.390, with a 95% CI of 0.315 to 0.460.

#### 3.6.2 Meta-analysis of the impact of exercise self-efficacy on physical activity in the elderly

Among the 19 studies included, six employed multiple regression analysis to provide effect sizes (standardized coefficients *β*) of exercise self-efficacy on physical activity in elderly individuals. The 95% confidence intervals were calculated via the following formula: 95% CI = β ± 1.96 × SE. A random-effects model was used to conduct the meta-analysis on the standardized coefficients β and their 95% CIs.

**Figure 3:**
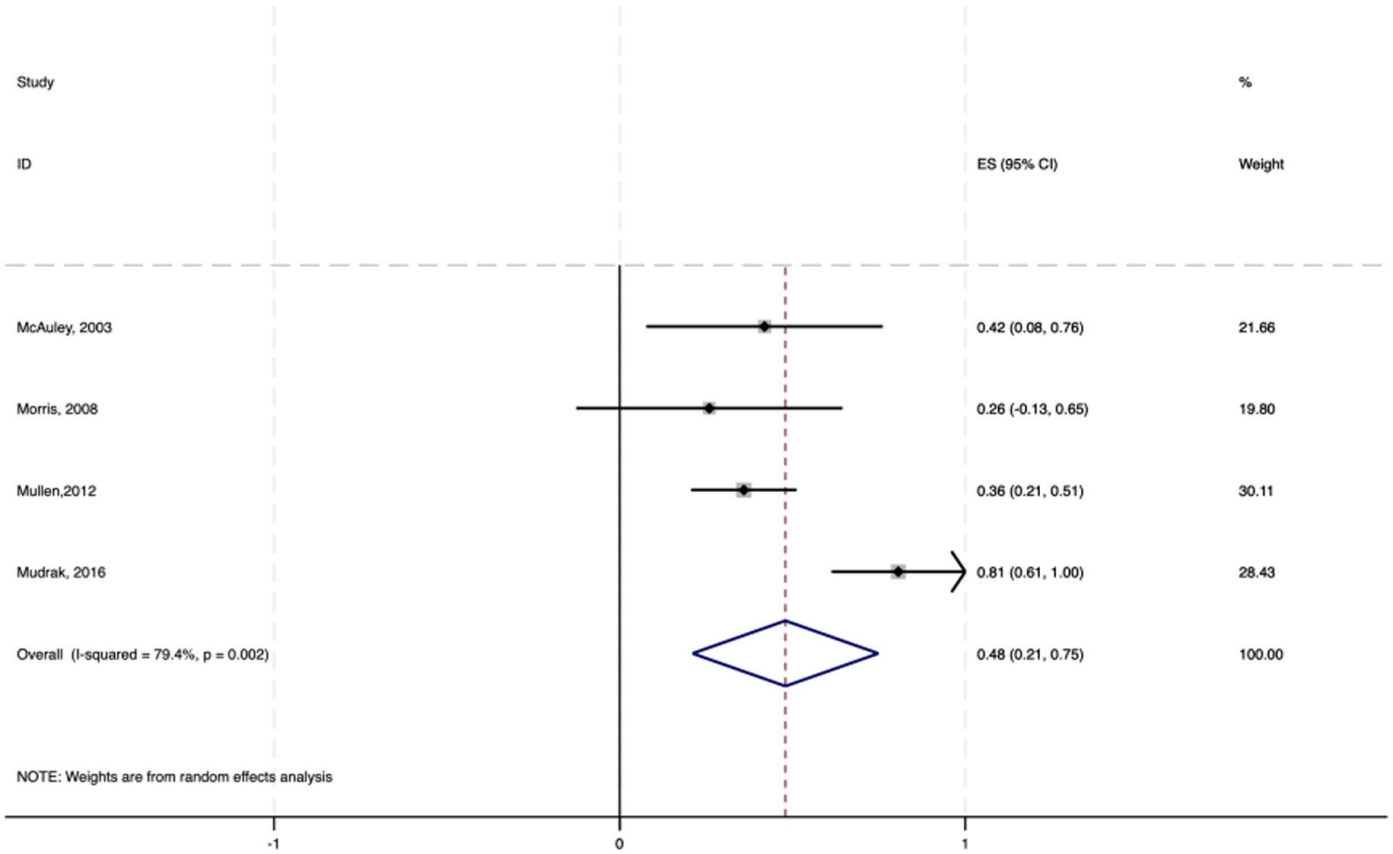
Forest plot of the effect of physical activity on exercise self-efficacy among elderly individuals.

The results indicate a pooled effect size of 0.386, with a 95% CI ranging from 0.221 to 0.551, suggesting a significant positive impact of exercise self-efficacy on physical activity in elderly individuals. The heterogeneity test yielded a chi-square value of 31.00 with 5 degrees of freedom and a *p* value less than 0.0001, indicating significant heterogeneity among studies. The I-squared value of 83.9% suggested that 83.9% of the variability in effect sizes was due to heterogeneity between studies. Furthermore, the z test for an effect size of zero yielded a value of 4.58 with a p value less than 0.0001, further confirming the statistical significance of the findings (see Figure 3).

In summary, exercise self-efficacy has a moderate positive impact on physical activity in elderly individuals, with a standardized coefficient of 0.386. This implies that for every standard deviation increase in exercise self-efficacy, there is an average increase of 0.386 standard deviation units in physical activity levels among elderly individuals.

#### 3.6.3 Meta-analysis of the impact of physical activity on exercise self-efficacy in elderly individuals

Among the 20 studies included, four utilized structural equation modeling (SEM) to assess the impact of physical activity on exercise self-efficacy in elderly individuals, providing effect sizes in the form of path coefficients (*γ*). Standard errors (SE) and 95% confidence intervals (95% CI) were calculated via path coefficients and sample sizes with the following formulas: SE = γ/√n, 95% CI = γ ± 1.96 × SE. A random-effects model was employed for the meta-analysis of the path coefficients γ and their 95% CIs.

**Figure 4:**
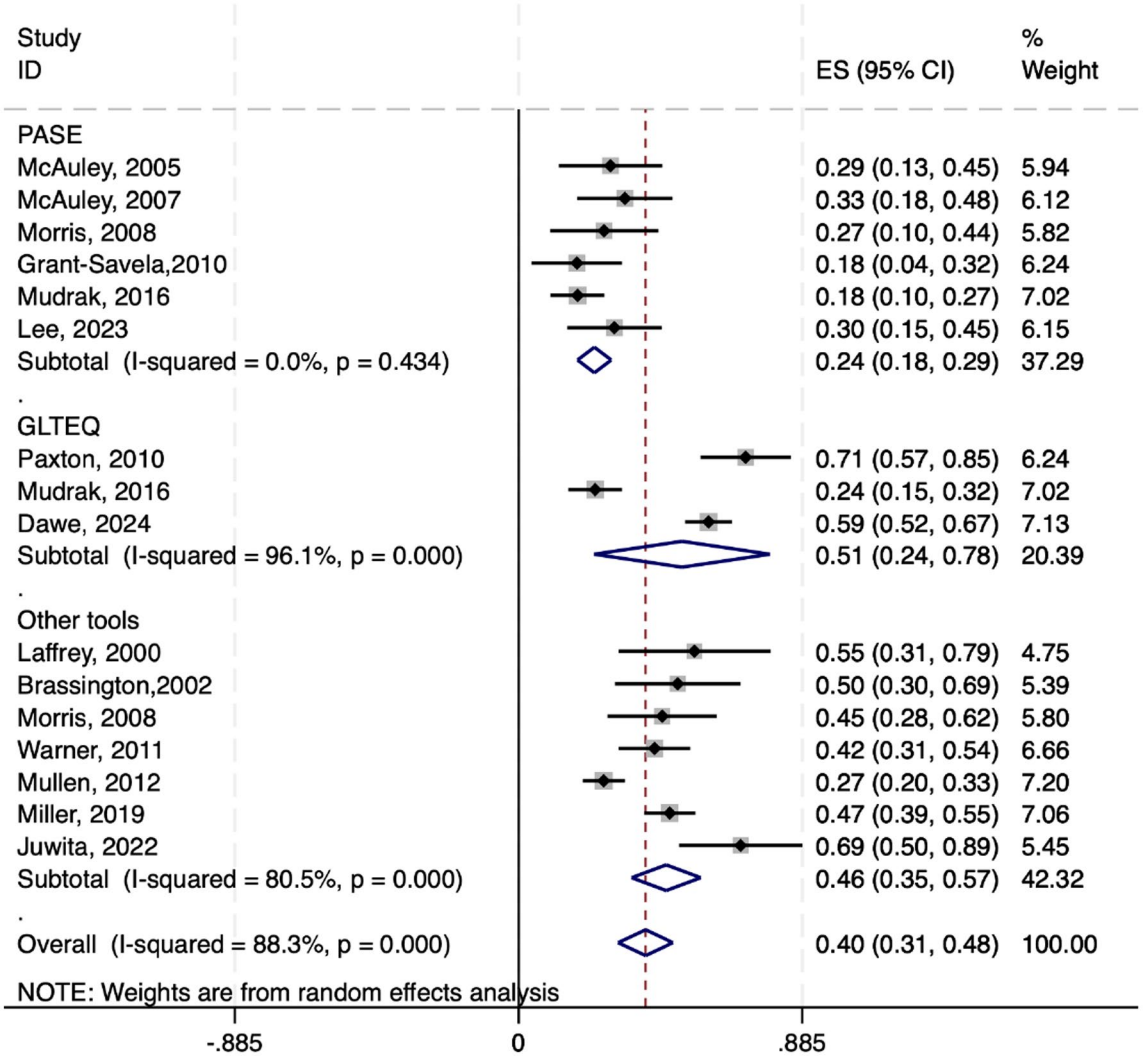
Forest plot of the subgroup analysis based on different physical activity assessment tools.

The results revealed a pooled effect size of 0.481, with a 95% CI ranging from 0.211 to 0.750, indicating a significant positive impact of physical activity on exercise self-efficacy in elderly individuals. The heterogeneity test yielded a chi-square value of 14.59 with 3 degrees of freedom and a *p* value less than 0.01, suggesting significant heterogeneity among studies. The I-squared value of 79.4% indicates that 79.4% of the variability in effect sizes is due to heterogeneity between studies. Moreover, the z test for an effect size of zero yielded a value of 3.49 with a p value less than 0.0001, further confirming the statistical significance of the findings (see Figure 4).

In summary, physical activity in elderly individuals has a moderate positive impact on exercise self-efficacy, with an average path coefficient of 0.481. These findings suggest that an increase in the level of physical activity among the elderly is associated with a corresponding increase in their exercise self-efficacy, with an estimated increase of approximately 48.1% in the activity level.

### 3.7 Subgroup analysis

To investigate the impacts of different physical activity assessment tools on the results, a subgroup analysis was conducted. Specifically, the data were divided into three subgroups on the basis of the use of the PASE, GLTEQ, and other assessment tools. These tools included the PASE (6studies), GLTEQ (3 studies), and other tools, such as the Actigraph accelerometer (7studies), which were used to analyze the associations between physical activity and exercise self-efficacy in elderly individuals.

The subgroup analysis revealed that the pooled effect size for the PASE was 0.237 (95% CI: 0.184 to 0.290), that for the GLTEQ was 0.510 (95% CI: 0.236 to 0.785), and that for the other assessment tools was 0.461 (95% CI: 0.353 to 0.569). The *p* values for all three subgroups were less than 0.001, indicating a significant effect across studies. Heterogeneity tests revealed that the I-squared values for the PASE, GLTEQ, and other tools were 0.0, 96.1, and 80.5%, respectively, indicating significant heterogeneity within each subgroup (see Figure 4).

After converting the Fisher’s z scores and 95% CIs back to correlation coefficients and their 95% CIs, the correlation coefficient between PASE and exercise self-efficacy was found to be 0.232 (95% CI: 0.182 to 0.282), that between the GLTEQ score and exercise self-efficacy was 0.470 (95% CI: 0.231 to 0.656), and that between other tools and exercise self-efficacy was 0.431 (95% CI: 0.339 to 0.514). Therefore, the average correlation coefficients between the PASE, GLTEQ, and other tools with exercise self-efficacy were 0.232, 0.470, and 0.431, respectively.

### 3.8 Publication Bias assessment

**Figure 5:**
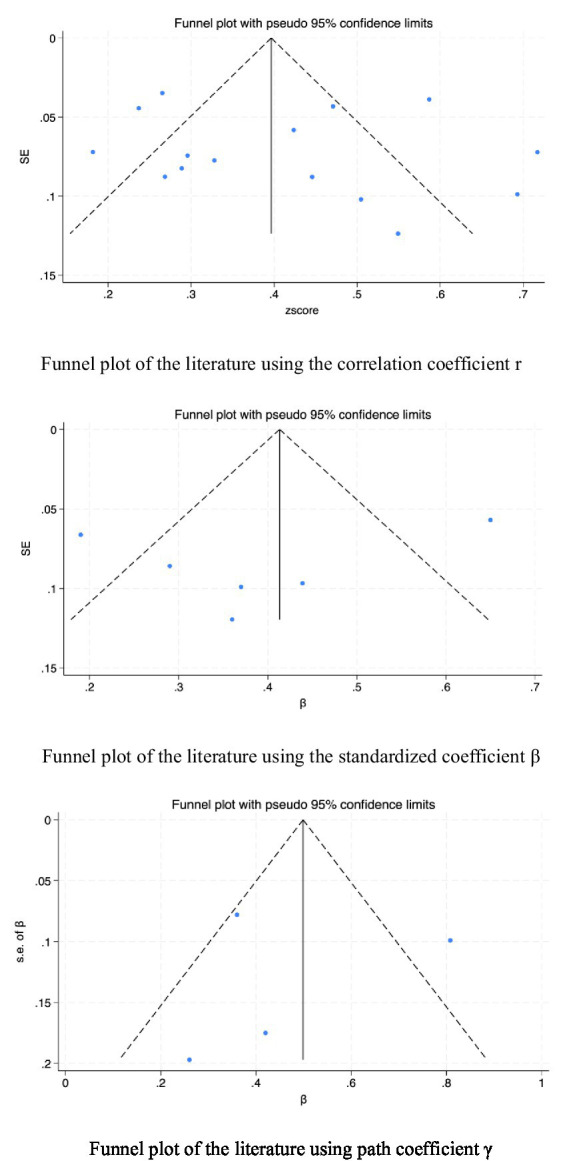
Funnel plot analysis of the association between exercise self-efficacy and physical activity among elderly individuals.

Publication bias in the included studies was examined via funnel plots and Egger’s test. The symmetry of the funnel plots (Figure 5) and the intercept coefficient *p* values from Egger’s test (Table 4) for different types of effect sizes (statistical correlation data r, standardized coefficient *β*, and path coefficient *γ*) were 0.613, 0.471, and 0.517, respectively. These findings suggest that there is no publication bias in the statistical correlation data r. Additionally, there is insufficient evidence to indicate the presence of small sample effects or publication bias for the standardized coefficient β and path coefficient γ.

**Table 4:** Results of the publication bias test.

Data Type	*P* value of slope	*P* value of bias	Is there publication bias?
Correlation data r	*p* = 0.012	*p* = 0.613	No
Standardized coefficient β	*p* = 0.116	*p* = 0.471	No
Path coefficient γ	*p* = 0.137	*p* = 0.517	No

## 4 Discussion

By adhering to PRISMA guidelines, it aims to comprehensively and transparently outline the current evidence regarding this relationship in older adults. Our analysis of 19 high—quality studies (both cross—sectional and longitudinal) shows a significant positive link between exercise self—efficacy and physical activity. Exercise self—efficacy positively affects physical activity and vice versa. These results highlight their bidirectional relationship, offering a theoretical basis for promoting physical activity and enhancing the quality of life in older adults.

To further explore this association, we conducted subgroup analysis on the basis of different physical activity assessment tools, such as the PASE scale and GLTEQ. The findings indicated a positive correlation between physical activity and exercise self-efficacy within all subgroups, validating the effectiveness of the assessment tools. However, heterogeneity tests among subgroups highlighted significant differences between studies, which may be attributed to variations in sample characteristics, research design, and measurement tools. Previous research has highlighted the limitations of traditional assessment tools. For instance, McAuley et al. (2005) and Harris et al. (2009) pointed out these limitations and recommended the use of more refined evaluation methods.

Furthermore, we performed a publication bias assessment, with neither funnel plots nor Egger’s test indicating bias, thereby enhancing the reliability of our results. These findings provide empirical evidence for understanding the role of exercise self-efficacy in promoting physical activity among elderly individuals and offer guidance for future research directions and methodologies.

This review makes an important breakthrough compared to previous studies. Early studies often focused on clinical populations (e.g., individuals with chronic obstructive pulmonary disease or diabetes) or younger groups. In contrast, this review demonstrates that the bidirectional relationship between self-efficacy and physical activity is also prevalent in healthy elderly populations and is not affected by disease-related factors. Unlike Medrano-Ureña et al. (2020), who emphasized a unidirectional path in adults, this review leverages longitudinal and structural equation modeling meta-analyses to provide more robust empirical support for Bandura (1997) interactional theory. It underscores the importance of designing interventions that target both self-efficacy and physical activity simultaneously.

### 4.1 The specificity and objectivity of the assessment tools

The study employed multiple tools to measure physical activity and exercise self-efficacy, including the Physical Activity Scale for the Elderly (PASE) and the Godin Leisure Time Exercise Questionnaire (GLTEQ). These tools differ significantly in measurement precision, time frame, and subjectivity. For instance, the PASE focuses more on daily activities, while the GLTEQ emphasizes leisure-time physical activity. Despite their respective advantages and limitations, both tools are widely used in their respective fields and have established reliability and validity.

To further verify this association, we performed a subgroup analysis across different tools. All subgroups showed a positive correlation, indicating these tools’ validity in measuring the relevant variables. Yet, the heterogeneity testing between subgroups revealed significant study—to—study differences. This might stem from the tools’ specificity—related biases, such as those between self—reported and objectively measured data. McAuley et al. (2005) and Harris et al. (2009) highlighted the limitations of traditional tools. By incorporating multidimensional assessment tools like SEM and accelerometers, this study partly addresses their criticisms. However, there’s still a need to develop more specific and objective tools tailored to the physiological and psychological characteristics of elderly individuals to reduce bias and enhance measurement precision.

### 4.2 Mechanisms underlying the association between physical activity and exercise self-efficacy in elderly individuals

The meta-analysis of correlation coefficients revealed a moderate positive relationship between physical activity and exercise self-efficacy among the elderly (average correlation coefficient r = 0.412, *p* < 0.001), suggesting that elderly individuals who actively engage in physical activity tend to have greater exercise self-efficacy. This finding aligns with previous review studies on patients with chronic obstructive pulmonary disease (COPD) (Selzler et al., 2020) and diabetes mellitus (DM) (Hamidi et al., 2022), which also revealed a link between increased self-efficacy and increased levels of physical activity.

Additionally, a meta—analysis of six studies using multiple regression analysis showed a significant positive link between exercise self—efficacy and physical activity levels in the elderly (average standardized coefficient *β* = 0.386, *p* < 0.001). The results of this study underscore the pivotal role of exercise self-efficacy in promoting physical activity in elderly individuals, which is consistent with Bandura (1977) self-efficacy theory. Elderly individuals with high exercise self-efficacy are more likely to participate in and adhere to physical exercise, as they believe that they can overcome challenges such as physical discomfort and poor weather. This positive mindset and behavioral pattern combination aids in overcoming physical and psychological barriers, thereby enhancing physical activity levels. This perspective is supported by several studies, including those by Lee and Fan (2023) and Perkins et al. (2008), which indicate that exercise self-efficacy is a significant factor in promoting the participation of elderly individuals in physical and social activities.

Furthermore, the meta-analysis of structural equation model data revealed a significant relationship between increased physical activity and increased exercise self-efficacy in elderly individuals (average path coefficient of 0.481, *p* < 0.001). These findings suggest that physical activity can enhance exercise self-efficacy in elderly individuals, which may be related to the positive health impacts of physical activity. Research by Mullen et al. (2012) shows that physical activity not only improves physical function but also reduces functional limitations, thereby promoting the development of exercise self-efficacy. Paxton et al. (2010) also highlighted the role of mental health in this process.

**Figure 6:**
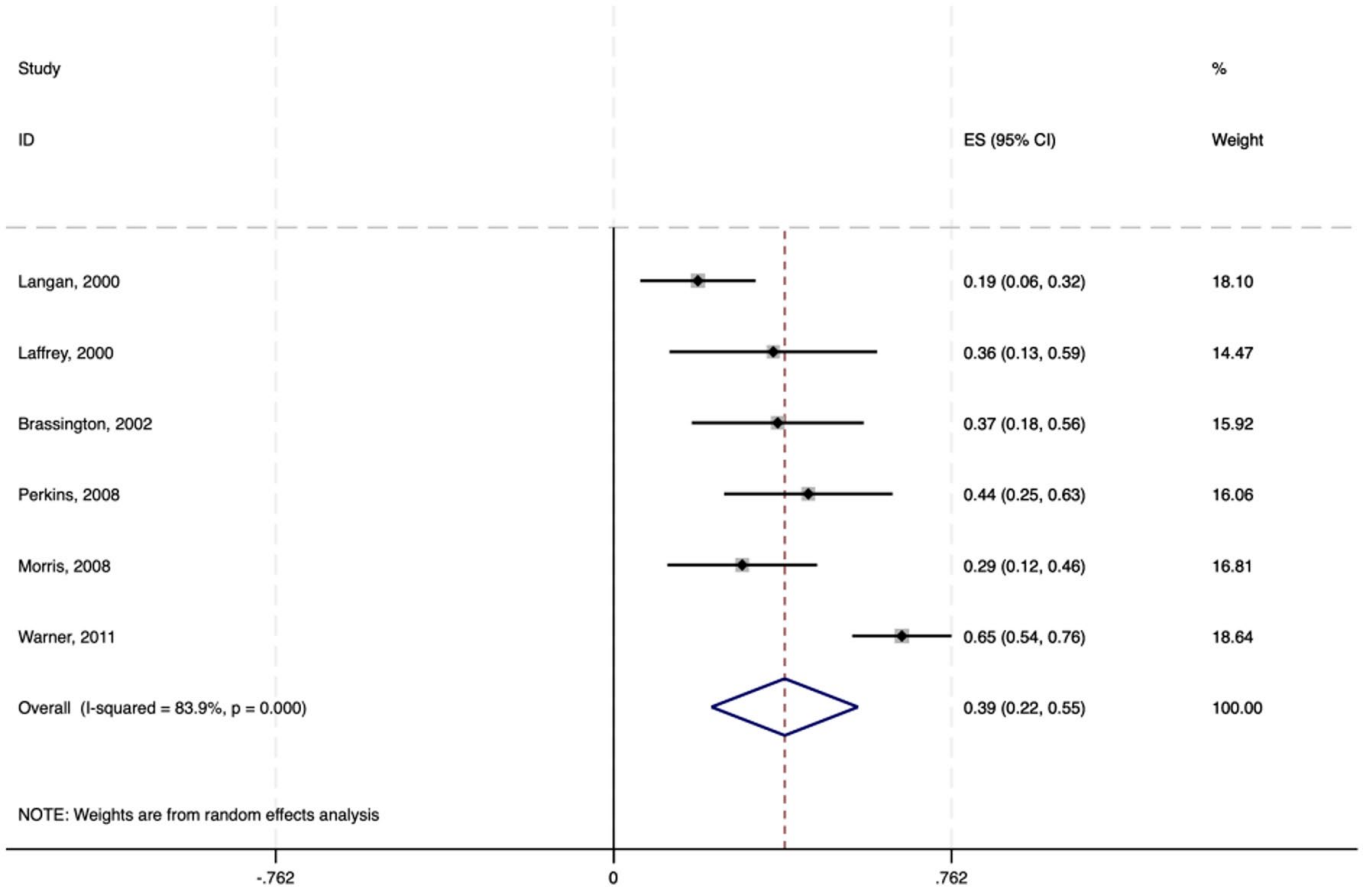
Forest plot of the effect of exercise self-efficacy on physical activity among elderly individuals.

Also, the slight asymmetry in Figures 2, 6 may hint at publication bias. But Egger’s test had a *p* value > 0.05, indicating no significant bias. This minor asymmetry might stem from study diversity, varying sample sizes, and different measurement tools. Overall, there is a positive feedback loop between physical activity and exercise self-efficacy in elderly individuals: physical activity enhances self-efficacy, which in turn promotes more physical activity. This bidirectional relationship suggests that interventions to promote physical activity in the elderly should consider both enhancing exercise self-efficacy and encouraging active participation in physical activities to create a virtuous cycle. These findings provide important theoretical and practical support for improving the quality of life of elderly individuals.

### 4.3 Other factors associated with physical activity and exercise self-efficacy

The association between physical activity and exercise self-efficacy in elderly individuals is influenced by various factors, including social support, the neighborhood environment, cultural differences, and occupational involvement. Social support, a frequently cited factor in the literature, plays a significant role in the physical activity participation and exercise self-efficacy of elderly individuals. Research by Orsega-Smith et al. (2007) indicates that social support from friends is significantly associated with perceived physical self-efficacy and long-term physical activity (LTPA) among elderly individuals. Warner et al. (2011) further suggested that the interaction between exercise self-efficacy and social support enhances the frequency and persistence of physical exercise in elderly individuals.

The neighborhood environment is also a crucial factor. Morris et al. (2008b) reported that satisfaction with the neighborhood environment and perceived functional status are key predictors of exercise self-efficacy and subsequent physical activity behaviors. Lee and Fan (2023) emphasized the interactive relationship between the neighborhood environment and exercise self-efficacy, confirming its positive impact on promoting physical activity.

Cultural differences cannot be overlooked. Dawe et al. (2024) revealed variations in the associations between exercise self-efficacy and physical activity across different cultural contexts. Occupational background is another significant factor. Juwita and Damayanti (2022) reported that elderly individuals who continue to be occupationally involved exhibit higher levels of physical activity and confidence in their exercise capabilities.

### 4.4 Limitations and recommendations for future research

This study offers robust evidence for the relationship between exercise self-efficacy and physical activity, yet some limitations warrant attention. First, the majority of the included studies focused on the elderly population in the United States. This geographic concentration may affect the generalizability of the findings to other regions. The social and cultural context in the United States, such as the emphasis on individualism and the availability of specific physical activity resources, might influence how exercise self-efficacy is developed and how it relates to physical activity behaviors. In contrast, elderly individuals in European or Asian countries may be influenced by different cultural values, community structures, and healthcare systems. For instance, in some Asian cultures, there may be a greater emphasis on group-based physical activities and family support, which could interact with exercise self-efficacy in unique ways. Therefore, future research should extend the investigation to more diverse geographical and cultural settings to enhance our understanding of the universal and context-specific factors that shape the relationship between exercise self-efficacy and physical activity in elderly populations worldwide.

Additionally, since 2000, it’s been crucial to consider the evolving concepts and practices regarding physical activity in the elderly. During this time, there’s been a growing recognition that physical activity offers benefits beyond the physiological, emphasizing psychological factors like exercise self—efficacy. However, events like the COVID–19 pandemic may have significantly altered physical activity patterns and self—efficacy perceptions in the elderly. The pandemic restricted outdoor activities and changed social interactions, likely affecting how the elderly engage in physical activity and perceive their ability to do so. Future research could explore the specific impacts of such global events on the relationship between exercise self—efficacy and physical activity in the elderly, complementing the long—term trends summarized in this study.

The exclusion of the Embase database from this study may have limited the retrieval of articles published in biomedical journals. Future systematic reviews should include Embase to improve the comprehensiveness of evidence synthesis. The assessment tools used in this study have insufficient specificity and objectivity. For instance, physical activity is often measured via self—report tools like the PASE and GLTEQ, which are prone to subjective bias. Although accelerometer—based methods can offer more reliable data, their small sample sizes limit the representativeness of the findings. Future research should develop more specific and objective assessment tools to reduce bias and increase the sample size when using accelerometer—based methods, enhancing the representativeness and reliability of the results.

In our research, we define elderly individuals as those aged 60 and above, with an average age of 65 and above. This definition is in line with the WHO’s standard and aims to capture trends related to early—stage aging. However, this approach may include participants who have not yet experienced obvious functional decline. Although the requirement for an average age of 65 and above reduces the impact of younger outliers, future research could conduct more stratified analyses by narrowing the age range (e.g., 60–69 and 70—above). This would clarify how the relationship between exercise self—efficacy and physical activity changes across different stages of old age. The limitation of this study only selecting English literature may lead to the research scope not being comprehensive enough, failing to cover important research results in other languages, and thus affecting the universality of the research conclusions. In addition, since the conduct of the study may have certain regional and group characteristics, its results may not fully represent the characteristics of special regional groups. To this end, future research should consider including multilingual literature to ensure the comprehensiveness and universality of the research. At the same time, it is recommended to expand the sample range and increase attention to the elderly in special regions, so as to improve the representativeness and applicability of the research results.

Also, the assessment tools lack specificity and objectivity. For instance, physical activity is mainly measured via self-reported tools (e.g., PASE, GLTEQ), prone to subjective bias. While accelerometer-based measurements (as in Harris et al., 2009) offer more reliable data, their small sample sizes reduce representativeness. Moreover, excluding studies with fewer than 50 participants might introduce bias by removing extreme results. Future research should verify findings across different sample sizes and combine multiple measurement tools to minimize bias from a single instrument. Finally, this review excluded individuals with chronic illnesses, yet many older adults do have underlying conditions, which may affect the generalizability of the findings. Future research should further investigate the impact of chronic diseases on the relationship between exercise self-efficacy and physical activity to gain a more comprehensive understanding of the exercise behaviors of the elderly population.

### 4.5 Heterogeneity and its potential sources

The meta-analysis revealed substantial heterogeneity among the studies. Although the random-effects model accounted for between-study variability, the high heterogeneity warranted a more in-depth analysis of its sources. The primary potential factors are as follows:

#### 4.5.1 Differences in study designs

The included studies show marked methodological differences, comprising 13 cross-sectional and 6 longitudinal studies. Cross-sectional studies capture associations at a single time point, while longitudinal studies explore temporal relationships. For instance, McAuley et al. (2005) reported that correlations significantly weakened over time (*r* = 0.28 at 12 months, *r* = 0.09 at 60 months), which suggests that differences in study design may be a significant source of heterogeneity.

#### 4.5.2 Cultural and geographical differences

The studies cover several countries, including the US, Germany, South Korea, and Indonesia, which have significant differences in cultural norms and the accessibility of sports facilities. For example, Dawe et al. (2024) pointed out cultural differences in self—efficacy between participants from the US and Italy. Juwita and Damayanti (2022) emphasized that socioeconomic barriers in Indonesia might affect the elderly’s physical activity. These factors may influence the strength of the link between exercise self—efficacy and physical activity.

#### 4.5.3 Differences in measurement tools

The characteristics of physical activity assessment tools vary. They include self—report tools like the PASE and GLTEQ, and objective measurement tools such as the Actigraph accelerometer. Subgroup analysis shows that the pooled effect sizes for the PASE (*r* = 0.232) and GLTEQ (*r* = 0.470) differ significantly. This indicates that tool—specific biases (e.g., self—reported vs. objective measurements) may introduce heterogeneity. Similarly, exercise self—efficacy measurement tools also vary. Examples are the general—exercise ESE and the walking—specific WSE. These differences may further increase between—study variability.

#### 4.5.4 Disparities in sample characteristics

Differences in the age ranges (60–96 years), gender distribution (with a female percentage of 42–100%), and health status (such as working versus retired individuals) of study samples can moderate the strength of associations. For example, Morris et al. (2008b) focused exclusively on a female cohort, while Lee and Fan (2023) incorporated a mixed-gender sample, reflecting the diverse dynamics of different populations.

#### 4.5.5 Differences in statistical methods

Studies have employed a variety of analytical methods, including Pearson correlations, multiple regression, and structural equation modeling (SEM). Path coefficients derived from SEM [such as *γ* = 0.808 from Mudrak et al., 2016] are often larger than effect sizes from regression models. This might be because SEM can incorporate latent variables and measurement errors.

#### 4.5.6 Temporal and contextual changes

The time span of studies ranges from 2000 to 2024, which introduces temporal heterogeneity. Societal changes, like the COVID-19 pandemic, may have altered physical activity patterns and self-efficacy perceptions in later studies. However, the current dataset has insufficient exploration of such impacts.

This study offers strong evidence on the relationship between exercise self—efficacy and physical activity, but the following limitations should be noted. Firstly, high heterogeneity implies that unmeasured moderators (such as cultural norms and measurement tool biases) may mask the true effect size.Secondly, the studies are predominantly from Western countries (from the US and Europe),which limits the generalizability of the results to resource—poor regions.Thirdly, the reliance on self—reported physical activity measures (such as the PASE and GLTEQ) may introduce recall bias.Although accelerometer—based studies provide more objective data, their small sample sizes limit their representativeness.Future research should prioritize longitudinal designs,culturally adapted tools and stratified analyses to explore the sources of heterogeneity,and develop more precise and standardized measurement tools to enhance the reliability and comparability of the findings.

## 5 Conclusion

This study confirmed a significant positive correlation between exercise self-efficacy and physical activity in elderly individuals, with a moderate positive impact of exercise self-efficacy on physical activity levels among elderly individuals. Conversely, physical activity also positively influences exercise self-efficacy. These findings provide a theoretical basis for promoting physical activity among elderly individuals to increase their quality of life. Future research should further investigate the roles of various influencing factors and develop targeted intervention strategies to encourage more active engagement in physical activities among elderly individuals.
